# Bone Marrow Changes in Septic Shock: A Comprehensive Review

**DOI:** 10.7759/cureus.42517

**Published:** 2023-07-26

**Authors:** Nimmanagoti Nagaraju, Ashish Varma, Amar Taksande, Revat J Meshram

**Affiliations:** 1 Paediatrics, Jawaharlal Nehru Medical College, Datta Meghe Institute of Higher Education and Research, Wardha, IND

**Keywords:** therapeutic strategies, leukocyte production, immune dysregulation, hematopoiesis, septic shock, bone marrow

## Abstract

Septic shock is a life-threatening condition characterized by systemic inflammation resulting from a severe infection. Although the primary focus of sepsis research has traditionally been on the dysfunctional immune response, recent studies have highlighted the important role of bone marrow in the pathophysiology of septic shock. The bone marrow, traditionally regarded as the hematopoietic organ responsible for blood cell production, undergoes significant changes during sepsis, contributing to the overall immune dysregulation observed in this condition.

This comprehensive review aims to provide a detailed overview of the bone marrow changes associated with septic shock. It explores the alterations in the bone marrow microenvironment, hematopoietic progenitor cells, and the subsequent effects on leukocyte production and function. Key cellular and molecular mechanisms involved in bone marrow dysfunction during septic shock are discussed, including the dysregulation of cytokines, chemokines, growth factors, and signaling pathways. Furthermore, this review highlights the clinical implications of bone marrow changes in septic shock. It emphasizes the impact of altered hematopoiesis on immune cell populations, such as neutrophils, monocytes, and lymphocytes, and their role in the progression and outcome of sepsis. The potential prognostic value of bone marrow parameters and the therapeutic implications of targeting bone marrow dysfunction are also addressed. The review summarizes relevant preclinical and clinical studies to comprehensively understand the current knowledge of bone marrow changes in septic shock. The limitations and challenges of studying bone marrow in the context of sepsis are acknowledged, and future directions for research are proposed.

## Introduction and background

Septic shock is a life-threatening condition characterized by a dysregulated immune response to infection, leading to widespread inflammation, organ dysfunction, and high mortality rates. It is considered a medical emergency requiring immediate intervention and intensive care support. Despite advances in critical care management, septic shock remains a major global health challenge [1-3]. Septic shock arises from the complex interplay between invading pathogens, the immune system, and host responses. The initial infection triggers a cascade of events, including the release of pro-inflammatory mediators such as cytokines and the activation of various immune cells. The systemic inflammatory response can rapidly progress, leading to organ failure and circulatory collapse [4].

The bone marrow, the soft tissue found within the cavities of bones, plays a crucial role in the pathophysiology of septic shock. Traditionally recognized as the primary site for hematopoiesis, the production of blood cells, the bone marrow is also a dynamic immune organ involved in regulating and mobilizing immune cells. During septic shock, the bone marrow response becomes dysregulated, leading to significant changes in hematopoiesis and immune cell function [5].

This comprehensive review explores the intricate relationship between septic shock and bone marrow changes. By examining the alterations occurring within the bone marrow microenvironment and the consequences on hematopoietic function and immune cell homeostasis, we aim to provide a comprehensive understanding of the impact of septic shock on this vital organ. This review will delve into the knowledge surrounding bone marrow changes in septic shock, including the activation of hematopoietic stem cells, alterations in cell populations and differentiation, impaired blood cell production, disruption of the bone marrow microenvironment, and changes in immune cell function. Furthermore, we will discuss the diagnostic approaches and potential clinical implications of these bone marrow alterations and identify research gaps and future directions in this field.

## Review

Bone marrow anatomy and function

A Brief Overview of Bone Marrow Structure

Bone marrow, a specialized tissue, resides within the central cavities of long bones (e.g., femur, tibia) and flat bones (e.g., sternum, pelvis). It can be categorized into two main types: red marrow and yellow marrow. Red marrow is the key site of hematopoiesis, the process of blood cell production, while yellow marrow mainly consists of adipose tissue [6]. Red marrow is characterized by its rich vascularity and houses a variety of cells, including hematopoietic stem cells, progenitor cells, and stromal cells that provide support. Within the bone marrow, specific regions known as hematopoietic niches play a vital role in creating the microenvironment necessary to develop, differentiate, and mature blood cells [7]. These niches provide the necessary signals and cellular interactions to facilitate the growth and specialization of hematopoietic cells.

The hematopoietic niches within the red marrow create a dynamic and organized network that fosters the production of various blood cell lineages. Hematopoietic stem cells give rise to progenitor cells, which differentiate into specific cell types. Erythropoiesis occurs in specialized niches dedicated to red blood cell production; myelopoiesis contributes to the development of myeloid cells (e.g., neutrophils, monocytes); and lymphopoiesis supports the maturation of lymphoid cells (e.g., T cells, B cells). The intricate organization and cellular composition of bone marrow provide the foundation for hematopoiesis and immune cell development. Understanding the structure and functionality of bone marrow is essential to comprehending the impact of septic shock on this vital tissue [7].

Normal Bone Marrow Function

The bone marrow serves as the primary site for the production of all blood cells, including red blood cells (erythrocytes), white blood cells (leukocytes), and platelets (thrombocytes). This dynamic process, known as hematopoiesis, is tightly regulated and involves a complex interplay of cytokines, growth factors, and signaling pathways [8].

At the core of hematopoiesis are hematopoietic stem cells (HSCs), multipotent cells residing within the bone marrow. Hematopoietic stem cells can self-renew, maintaining a constant pool of stem cells and differentiating into progenitor cells. These progenitor cells, in turn, undergo further differentiation and specialization into specific cell lineages. Erythropoiesis leads to the production of red blood cells; myelopoiesis gives rise to a range of myeloid cells such as neutrophils and monocytes; and lymphopoiesis leads to the generation of lymphoid cells, including T cells and B cells [9].

In addition to its role in hematopoiesis, the bone marrow plays a crucial role in the development and maturation of immune cells. It provides a nurturing environment for lymphoid progenitor cells, facilitating their differentiation into functional immune cells. Within the bone marrow, B lymphocytes mature, acquiring their antigen receptor repertoire and becoming fully functional. T lymphocytes, on the other hand, initially originate from bone marrow-derived progenitor cells but migrate to the thymus for further development and maturation [10].

Hematopoiesis and Immune Cell Development

Hematopoiesis and immune cell development within the bone marrow are complex processes regulated by intricate cellular and molecular interactions. The bone marrow provides specialized niches composed of stromal cells, endothelial cells, and extracellular matrix components, which play crucial roles in supporting hematopoietic stem cells (HSCs) [11].

Within these niches, HSCs are maintained, proliferate, and undergo differentiation into various hematopoietic lineages. The communication between HSCs and the surrounding microenvironment is facilitated by critical signals provided by stromal cells, including growth factors, cytokines, and adhesion molecules. These signals regulate the balance between self-renewal and differentiation in HSCs, ensuring the continuous production of mature blood cells.

Cytokines, growth factors, and transcription factors also play key roles in guiding hematopoietic differentiation and lineage commitment. Cytokines, such as erythropoietin, granulocyte colony-stimulating factor (G-CSF), and thrombopoietin, influence the production and maturation of specific blood cell types. For example, erythropoietin stimulates the differentiation of erythroid progenitors into red blood cells, while G-CSF promotes the development of granulocytes, and thrombopoietin regulates platelet production. Transcription factors, including GATA-binding factor 1 (GATA-1) and PU.1 (also known as spi-1 proto-oncogene SPI1), are involved in the lineage-specific differentiation of hematopoietic progenitor cells, directing their commitment towards specific cell lineages [12].

In addition to hematopoiesis, the bone marrow is critical for developing immune cells. B lymphocytes undergo V(D)J recombination, where gene segments encoding immunoglobulins are rearranged, generating a diverse repertoire of B cell receptors (BCRs) capable of recognizing a wide range of antigens. This diversity is crucial for the adaptive immune response and the ability to recognize and eliminate pathogens. T lymphocytes, on the other hand, undergo positive and negative selection in the thymus, ensuring the development of functional T cell receptors (TCRs) while eliminating self-reactive T cells [13].

Pathophysiology of septic shock

Definition and Causes of Septic Shock

Septic shock is a grave and life-threatening condition characterized by an uncontrolled immune response to an infection. It represents the most severe manifestation of sepsis, a systemic inflammatory response syndrome caused by microorganisms or toxins in the bloodstream or other tissues. The causative agents of septic shock can include a wide range of microorganisms, such as bacteria, viruses, fungi, and parasites [14].

The onset of septic shock occurs when an initial infection triggers a series of complex events within the immune system. This includes recognizing pathogen-associated molecular patterns (PAMPs) by pattern recognition receptors (PRRs) on immune cells. The recognition of PAMPs leads to the release of pro-inflammatory mediators, broadly categorized into cytokines and chemokines. Cytokines, such as tumor necrosis factor-alpha (TNF-α) and interleukin-1, are essential pro-inflammatory signaling molecules crucial in initiating and amplifying the immune response. On the other hand, chemokines, another subset of pro-inflammatory mediators, also contribute significantly to the immune response by orchestrating the movement and migration of immune cells to the site of infection or inflammation [15].

Some examples of chemokines, including molecules such as CXCL8 (also known as interleukin-8), CCL2 (also known as monocyte chemoattractant protein-1), and CXCL10 (also known as interferon gamma-induced protein 10) facilitate the recruitment and activation of various immune cells, enabling an efficient immune response to combat the infection. However, in septic shock, this immune response becomes dysregulated and loses balance, resulting in an exaggerated and uncontrolled inflammatory reaction [15]. It is important to note that while cytokines and chemokines are crucial for initiating and coordinating the immune response, their dysregulated release can contribute to the pathogenesis of septic shock [15].

Overview of the Inflammatory Response

The inflammatory response is a complex biological process to eliminate invading pathogens and initiate tissue repair. It involves the activation of various immune cells and the production of inflammatory mediators. However, the inflammatory response in septic shock becomes dysregulated, leading to widespread tissue damage and organ dysfunction [16].

The cascade of events in the inflammatory response involves the activation of signaling pathways such as the nuclear factor-kappa B (NF-κB) pathway and the mitogen-activated protein kinase (MAPK) pathway. These pathways trigger the transcriptional upregulation of pro-inflammatory cytokines, chemokines, and adhesion molecules. Pro-inflammatory cytokines, including tumor necrosis factor-alpha (TNF-α), interleukin-1 (IL-1), and interleukin-6 (IL-6), play crucial roles in initiating and amplifying the immune response by recruiting and activating immune cells at the site of infection [17].

However, the balance between pro-inflammatory and anti-inflammatory processes is disrupted in septic shock. Alongside the excessive release of pro-inflammatory mediators, anti-inflammatory mechanisms, such as the release of anti-inflammatory cytokines (e.g., interleukin-10) and the activation of regulatory T cells, attempt to counterbalance the inflammatory response. Unfortunately, this counterbalance is insufficient in septic shock, leading to an exaggerated and sustained inflammatory state [18].

The dysregulated inflammatory response in septic shock can have severe consequences. It can result in endothelial dysfunction, increased vascular permeability, and microcirculatory impairment. These alterations contribute to tissue hypoperfusion, cellular oxygen deprivation, and organ dysfunction. The cardiovascular, respiratory, renal, gastrointestinal, and central nervous systems are among the organ systems affected by the dysregulated inflammatory response in septic shock.

Impact of Septic Shock on Multiple Organ Systems

Septic shock exerts profound effects on various organ systems throughout the body, primarily due to a dysregulated immune response and the release of inflammatory mediators. This dysregulation leads to endothelial dysfunction, increased vascular permeability, and microcirculation impairment. Consequently, tissue hypoperfusion and cellular oxygen deprivation occur, culminating in organ dysfunction and potential organ failure [19].

Septic shock can affect multiple organ systems, including the cardiovascular, respiratory, renal, gastrointestinal, and central nervous systems. Cardiovascular dysfunction is a hallmark of septic shock and is characterized by hypotension and reduced cardiac output, resulting in inadequate tissue perfusion. Acute respiratory distress syndrome (ARDS) can develop due to increased lung permeability and impaired gas exchange, further compromising respiratory function. The kidneys can suffer from sepsis-induced injury; liver dysfunction can manifest as hepatic impairment; and gastrointestinal dysfunction may arise, leading to impaired motility and absorption [20].

The intricate interplay between the dysregulated immune response, the release of inflammatory mediators, and the resulting organ dysfunction underscores the importance of understanding the impact of septic shock on the bone marrow. The subsequent sections of this review will delve into the specific changes occurring within the bone marrow microenvironment, hematopoiesis, and immune cell dynamics in the context of septic shock [21].

Bone marrow changes in septic shock

Bone Marrow Response to Sepsis

The bone marrow mounts a response to septic shock, aiming to maintain hematopoiesis and mobilize immune cells to combat the infection. This response involves activating hematopoietic stem cells (HSCs) and alterations in cell populations and differentiation. In the presence of inflammatory signals and cytokines, HSCs, which reside in specialized niches in the bone marrow, are activated. This activation triggers a cascade of events leading to enhanced proliferation and differentiation of HSCs. This response aims to replenish the immune cell pool needed to mount an effective immune response against the invading pathogens [22]. By activating HSCs, the bone marrow strives to maintain a sufficient supply of immune cells throughout septic shock. Septic shock also disrupts the balance of cell populations within the bone marrow. One prominent alteration is the expansion of myeloid progenitor cells and a shift towards myelopoiesis, the production of myeloid cells. This results in increased production of neutrophils and monocytes, which are crucial components of the innate immune response against infections [23].

The bone marrow prioritizes the generation of these myeloid cells to enhance the initial defense against the infection. Conversely, lymphopoiesis, the production of lymphoid cells, may be impaired in septic shock. This can lead to a relative decrease in lymphocytes, including T cells and B cells, which play essential roles in adaptive immune responses [23]. The imbalance between myelopoiesis and lymphopoiesis reflects the immune system's emphasis on generating cells involved in the immediate and rapid response to infection. The bone marrow response to septic shock illustrates its dynamic adaptability in the face of an infectious insult. By activating HSCs and altering cell populations and differentiation, the bone marrow strives to maintain immune cell homeostasis and support the immune response during septic shock. Further research is needed to uncover the precise mechanisms underlying these bone marrow responses and their implications for immune function in septic shock.

Hematopoietic Dysfunction in Septic Shock

Impaired production of blood cells: In septic shock, despite the activation of hematopoietic stem cells, there is often a disruption in the normal processes of hematopoiesis, resulting in hematopoietic dysfunction. The excessive inflammatory response, endothelial dysfunction, and tissue hypoperfusion that characterize septic shock can harm blood cell production. Consequently, there may be a decrease in the overall production of blood cells, including red blood cells (erythrocytes), white blood cells (leukocytes), and platelets. This hematopoietic insufficiency contributes to the development of common hematological abnormalities observed in septic shock patients, such as anemia, leukopenia, and thrombocytopenia [24].

Disruption of bone marrow microenvironment: The bone marrow microenvironment supports hematopoiesis and maintains immune cell homeostasis. It comprises various components, including stromal cells, extracellular matrix, and signaling molecules. However, in septic shock, the dysregulated immune response and the release of pro-inflammatory cytokines can disrupt the delicate balance of the bone marrow microenvironment. The inflammatory milieu alters the interaction between hematopoietic cells and the supporting stromal cells, leading to impaired proliferation, differentiation, and survival of hematopoietic progenitor cells [25]. The disruption of the bone marrow microenvironment and impaired hematopoiesis in septic shock contribute to the hematological abnormalities observed in affected individuals. The decreased production of red blood cells can lead to anemia, resulting in reduced oxygen-carrying capacity and tissue hypoxia. Leukopenia, characterized by low white blood cell counts, compromises the immune system's ability to fight off infections. Thrombocytopenia, a deficiency of platelets, affects normal blood clotting and can lead to an increased risk of bleeding. Understanding these hematopoietic dysfunctions is crucial in identifying potential therapeutic targets to mitigate the adverse effects of septic shock on bone marrow function.

Immune cell alterations in septic shock

Changes in Neutrophil and Monocyte Function

Neutrophils and monocytes play pivotal roles in the innate immune response and are significantly impacted by septic shock. In sepsis, these key immune system components can undergo functional alterations that compromise their ability to combat pathogens and maintain immune homeostasis [26] effectively. Neutrophils, or polymorphonuclear leukocytes, are highly specialized immune cells that respond rapidly to infection. However, in septic shock, neutrophils may exhibit impaired migration, reducing their ability to migrate to sites of infection. This impairment in neutrophil migration hinders their timely arrival at the site of infection, diminishing their capacity to clear pathogens effectively.

In septic shock, neutrophils may experience decreased phagocytic capacity, limiting their ability to engulf and eliminate invading microorganisms. Furthermore, their ability to generate reactive oxygen species, crucial for microbial killing, can be impaired. As a result, neutrophils become less effective in neutralizing pathogens, allowing them to persist and contribute to the ongoing inflammatory response. Monocytes, another essential component of the innate immune system, can also undergo functional alterations in septic shock. These alterations can include aberrant cytokine production, leading to an imbalance in the inflammatory response. Monocytes may produce excessive amounts of pro-inflammatory cytokines, contributing to hyperinflammation. Conversely, they may exhibit reduced production of anti-inflammatory cytokines, impairing the resolution of the inflammatory response [26].

Impact on Adaptive Immune Response

Septic shock significantly influences the adaptive immune response, specifically affecting T and B lymphocytes, key players in the immune defense against pathogens. These alterations in adaptive immune cell function contribute to the immune dysregulation observed in septic shock [27]. T lymphocytes, including CD4+ helper T cells and CD8+ cytotoxic T cells, are critical in coordinating cellular immune responses. In septic shock, T cells may demonstrate reduced proliferation and impaired production of cytokines, such as interferon-gamma and interleukin-2. These functional impairments can weaken the T cell-mediated immune response, compromising the clearance of pathogens and the regulation of immune reactions.

B lymphocytes, responsible for antibody production and humoral immune responses, can also be affected in septic shock. B cells may exhibit functional abnormalities, such as impaired antibody production and dysregulated B cell receptor signaling. These dysfunctions may result in decreased antibody levels and altered antibody specificity, diminishing the effectiveness of the humoral immune response against pathogens. The impact of septic shock on adaptive immune cell function has broad implications for the overall immune response and the ability to mount an effective defense against invading pathogens. The dysregulated immune response observed in septic shock, characterized by excessive inflammation and immune suppression, can further contribute to the impaired adaptive immune response.

Understanding the bone marrow changes in septic shock provides valuable insights into the dysregulation of hematopoiesis, immune cell dynamics, and the overall immune response. By unraveling the mechanisms underlying these changes, developing strategies to mitigate immune dysregulation and improve patient outcomes is possible. In the subsequent sections, we will delve into the diagnostic approaches for assessing bone marrow function, discuss the clinical implications of these bone marrow alterations in septic shock, and identify potential therapeutic strategies that target bone marrow dysfunction [28]. 

Diagnostic approaches

Laboratory Tests for Assessing Bone Marrow Function

Complete blood count (CBC): The CBC is a commonly used test that provides information on the overall blood cell counts, including red blood cells, white blood cells, and platelets. Alterations in these parameters, such as anemia (low red blood cell count), leukopenia (low white blood cell count), or thrombocytopenia (low platelet count), can indicate potential bone marrow dysfunction in septic shock. These abnormalities may suggest inadequate production or increased destruction of blood cells [29].

Peripheral blood smear: Examination of a peripheral blood smear allows for evaluating red blood cell morphology, white blood cell differentials, and platelet abnormalities. This microscopic analysis provides insights into potential bone marrow pathology, such as abnormal cell shapes, sizes, or inclusions. Identifying abnormal cells, such as blasts or dysplastic cells, can indicate underlying bone marrow dysfunction [30].

Bone marrow aspiration and biopsy: Bone marrow aspiration and biopsy are invasive procedures performed to assess the bone marrow microenvironment and hematopoietic cell morphology directly. During the aspiration, a small sample of bone marrow is obtained for analysis. The biopsy involves the extraction of a bone marrow core sample. These procedures help evaluate various aspects of bone marrow function, including cellularity, abnormal cells or infiltrates, and dysplastic changes. They provide critical information about the structural and cellular composition of the bone marrow, aiding in diagnosing and characterizing bone marrow disorders in septic shock [31].

Flow cytometry: Flow cytometry is a powerful technique used to analyze the phenotypic characteristics of different cell populations. In the context of bone marrow assessment, flow cytometry can aid in the identification and enumeration of specific cell types within the bone marrow, including hematopoietic stem cells, immune cell subsets, and abnormal cell populations. It allows for the detection of surface markers and intracellular proteins, providing insights into bone marrow cells' differentiation patterns and functional properties. Flow cytometry is particularly useful in assessing aberrant immune cell populations or identifying rare cell subsets in septic shock [32].

Imaging Techniques for Evaluating Bone Marrow Changes

Imaging techniques are valuable in providing additional information on bone marrow changes and alterations in the bone marrow microenvironment in septic shock. Two specific imaging modalities frequently used for this purpose are magnetic resonance imaging (MRI) and positron emission tomography (PET) scans [33].

Magnetic resonance imaging (MRI): An MRI is a non-invasive imaging technique that utilizes magnetic fields and radio waves to generate detailed images of the body's internal structures. In septic shock, MRI can assess various aspects of the bone marrow, including composition, cellularity, and infiltration. By analyzing the signal intensity of the bone marrow, MRI can identify changes that may indicate underlying pathology. For example, alterations in signal intensity, such as focal lesions, edema, or areas of abnormal enhancement, can provide important clues to bone marrow abnormalities associated with septic shock. These findings can assist in diagnosing and monitoring bone marrow changes in septic shock patients [34].

Positron emission tomography (PET) scan: A PET scan involves the injection of a radiotracer, typically fluorodeoxyglucose (FDG), which is taken up by cells with high metabolic activity. PET scans are often combined with computed tomography (CT) to provide anatomical information along with metabolic data. In the context of bone marrow assessment in septic shock, PET scans can evaluate bone marrow metabolism and detect areas of increased metabolic activity. Increased FDG uptake in the bone marrow can indicate an inflammatory response or infection within the bone marrow microenvironment. This information can be particularly valuable in identifying bone marrow involvement in septic shock and providing insights into the inflammatory processes within the bone marrow [35].

Biomarkers for Septic Shock and Bone Marrow Dysfunction

C-reactive protein (CRP): C-reactive protein is an acute-phase protein commonly elevated in septic shock. It serves as a marker of systemic inflammation and can be used to assess the severity of the inflammatory response. Monitoring CRP levels over time can also help gauge the response to therapy and guide treatment decisions [36].

Procalcitonin (PCT): Procalcitonin levels have been extensively studied to indicate bacterial infection and sepsis severity. Elevated PCT levels are associated with the presence of an underlying bacterial infection and can aid in the differentiation of septic shock from other inflammatory conditions. Monitoring PCT levels can assist in guiding appropriate antimicrobial therapy [37].

Cytokines: The measurement of pro-inflammatory cytokines, such as tumor necrosis factor-alpha (TNF-α), interleukin-6 (IL-6), and interleukin-8 (IL-8), provides insights into the inflammatory response in septic shock. Elevated levels of these cytokines reflect a dysregulated immune response and can help assess the severity of systemic inflammation. Furthermore, monitoring cytokine levels can help identify patients at higher risk of developing complications and guide treatment strategies [38].

Hematopoietic growth factors: Hematopoietic growth factors, including erythropoietin (EPO), granulocyte colony-stimulating factor (G-CSF), and thrombopoietin (TPO), are involved in regulating hematopoiesis and blood cell production. Monitoring the levels of these growth factors can provide information about the bone marrow's ability to respond to the increased demand for blood cell production in septic shock. Abnormal levels may indicate hematopoietic dysfunction and impaired bone marrow response [39].

Clinical implications and prognosis

Association Between Bone Marrow Changes and Patient Outcomes

Hematopoietic dysfunction: Hematopoietic dysfunction, characterized by impaired production of blood cells such as anemia, leukopenia, and thrombocytopenia, has been associated with worse outcomes in septic shock patients. These hematopoietic abnormalities can harm patient health, including increased susceptibility to infections due to compromised immune function. Furthermore, the decreased production of red blood cells can contribute to tissue hypoxia and impaired wound healing, further exacerbating the clinical condition [40].

Immune dysregulation: Bone marrow changes in septic shock can lead to immune dysregulation, affecting the functionality of immune cells. Reduced activity of neutrophils and monocytes, crucial components of the innate immune response, hampers their ability to control the primary infection effectively. Altered adaptive immune responses, including impaired T and B lymphocyte function, further contribute to immune dysregulation in septic shock. This dysregulation of immune cell function can lead to increased susceptibility to secondary infections and the inability to adequately clear pathogens. As a result, it exacerbates organ dysfunction and contributes to poor clinical outcomes in septic shock patients [41].

Predictive Value of Bone Marrow Assessment in Septic Shock

Assessing bone marrow changes and dysfunction holds predictive value in septic shock by providing valuable insights into the inflammatory response, hematopoietic dysfunction, and immune cell dynamics. These assessments have the potential to identify patients at higher risk for adverse outcomes and guide clinical decision-making [42].

Prognostic indicators: Specific bone marrow alterations observed in septic shock, such as decreased hematopoietic activity, increased dysplasia, or abnormal cellularity, have been associated with increased mortality rates. Monitoring these indicators can aid in risk stratification and help predict patient outcomes. Patients with more severe bone marrow dysfunction may require more intensive interventions and closer monitoring [43].

Treatment response: Serial assessments of bone marrow function can serve as an essential tool to monitor the response to therapy in septic shock. Improvement in bone marrow function, as reflected by the normalization of cell counts and morphological changes, can indicate a favorable response to treatment. On the other hand, persistent abnormalities in bone marrow parameters may necessitate re-evaluation and consideration of alternative or adjunctive interventions to optimize patient management [44].

Potential Therapeutic Strategies Targeting Bone Marrow Dysfunction

Hematopoietic growth factors: The administration of hematopoietic growth factors, such as erythropoietin, G-CSF, or TPO, may stimulate blood cell production and potentially mitigate hematopoietic dysfunction. However, the use of these growth factors in septic shock remains controversial, and further studies are needed to define their optimal role and timing [45].

Immunomodulatory therapies: Modulating the dysregulated immune response in septic shock may positively impact bone marrow function. Targeted therapies, such as anti-inflammatory agents or immune checkpoint inhibitors, have shown promise in preclinical and early clinical studies. However, more research is required to determine their safety and efficacy in septic shock [46].

Supportive care: Providing supportive care measures, including appropriate antimicrobial therapy, hemodynamic support, and adequate nutrition, is crucial to managing septic shock. Optimizing these supportive interventions can indirectly improve bone marrow function and immune cell dynamics [47]. Future research efforts should focus on identifying novel therapeutic targets and strategies that specifically address bone marrow dysfunction in septic shock. Additionally, translational studies are needed to bridge the gap between experimental findings and clinical implementation, ultimately translating promising therapeutic approaches into improved patient outcomes.

Future directions and research gaps

Areas for Further Investigation

Mechanistic insights: Elucidating the underlying molecular and cellular mechanisms responsible for bone marrow alterations in septic shock is crucial. In-depth studies are needed to understand the signaling pathways, transcriptional regulation, and microenvironmental changes contributing to hematopoietic dysfunction and immune cell alterations.

Longitudinal studies: Conducting longitudinal studies that track bone marrow changes over time in septic shock patients can provide valuable insights into the temporal dynamics of hematopoiesis and immune cell dynamics. Long-term follow-up studies can help elucidate the recovery process and identify potential predictors of long-term outcomes.

Biomarkers: Identifying reliable biomarkers that accurately reflect bone marrow function and predict clinical outcomes in septic shock is essential. The discovery and validation of specific biomarkers associated with bone marrow alterations can aid in risk stratification, treatment monitoring, and prognostication.

Emerging Technologies and Techniques

Single-cell analysis: Single-cell RNA sequencing and mass cytometry allow for high-resolution analysis of individual cells within the bone marrow, providing insights into cell heterogeneity, lineage commitment, and functional alterations. These techniques can uncover novel cell subsets and transcriptional profiles associated with septic shock and guide targeted interventions.

Imaging modalities: Advanced imaging techniques, such as multiparametric MRI and PET-MRI, offer opportunities to non-invasively assess bone marrow composition, metabolism, and microenvironmental changes. Combining imaging with molecular probes or contrast agents can enhance the detection and characterization of bone marrow alterations in septic shock.

Organ-on-a-chip models: Utilizing organ-on-a-chip models that mimic the bone marrow microenvironment can provide a controlled experimental system for studying the interactions between immune cells, stromal cells, and pathogens. These models can help dissect the complex cellular and molecular mechanisms underlying bone marrow changes in septic shock.

Importance of Translational Research in Septic Shock

Therapeutic development: Translational research enables the translation of preclinical findings into novel therapeutic strategies targeting bone marrow dysfunction. Collaborations between basic scientists, clinicians, and industry partners are essential for developing and testing innovative interventions in clinical trials.

Precision medicine: Translational research can facilitate the identification of biomarkers and molecular signatures that predict treatment response and guide personalized therapeutic approaches in septic shock. Stratifying patients based on their specific bone marrow alterations can aid in tailoring interventions and optimizing outcomes.

Clinical decision support: Integrating translational research findings into clinical practice guidelines and decision support tools can improve the management of septic shock. Implementing algorithms that consider bone marrow changes and their implications can help guide therapeutic decisions, including selecting targeted therapies or supportive interventions.

The limitations and challenges in studying bone marrow in the context of sepsis

Studying bone marrow in the context of sepsis presents several limitations and challenges. The accessibility and invasiveness of obtaining bone marrow samples through procedures like aspiration and biopsy make it difficult to collect samples from critically ill septic shock patients. Additionally, sepsis is a dynamic condition with varying stages, making it challenging to capture the full spectrum of bone marrow changes at specific time points. Limited sample sizes and inter-individual variability hinder the ability to draw definitive conclusions or establish universal biomarkers. Furthermore, pre-existing conditions and comorbidities in septic shock patients can confound the interpretation of bone marrow changes specific to sepsis. Ethical concerns surrounding invasive procedures and potential patient risks also limit the scope of experimental studies. Translating findings from animal models to human clinical practice remains challenging, and the lack of standardized protocols for assessing bone marrow function adds to the complexity.

Moreover, sepsis is a multifactorial syndrome, making it difficult to isolate the specific effects of sepsis on the bone marrow. Despite these limitations, ongoing research, technological advancements, and collaborative efforts promise to overcome these challenges and deepen our understanding of bone marrow alterations in septic shock. Acknowledging and addressing these limitations is crucial for future advancements in diagnosis, prognostication, and targeted interventions.

## Conclusions

In conclusion, this comprehensive review highlights the significant findings regarding bone marrow changes in septic shock. The bone marrow response to sepsis involves activating hematopoietic stem cells, altering cell populations and differentiation, and shifting towards myelopoiesis. This dysregulation in hematopoiesis leads to impaired production of blood cells and disruption of the bone marrow microenvironment. Additionally, septic shock affects immune cell function, including changes in neutrophil and monocyte activity and compromised adaptive immune responses. These bone marrow alterations have important implications for clinical practice, including risk stratification, prognostication, and treatment decision-making in septic shock. Furthermore, further research is needed to understand the underlying mechanisms, develop targeted therapeutic strategies, and explore the predictive value of bone marrow assessment. By deepening our understanding of bone marrow changes, we can improve patient care and contribute to advancements in septic shock.
